# Aetiologies of Acute Complications in Autoimmune Rheumatologic Diseases: A Hospital-Based Cross-Sectional Study

**DOI:** 10.7759/cureus.35916

**Published:** 2023-03-08

**Authors:** Aparna P, Vaibhav Ingle, Abhishek Singhai, Sagar Khadanga, Rajnish Joshi, Saurabh Saigal, Ashwin Kotnis

**Affiliations:** 1 Internal Medicine, All India Institute of Medical Sciences, Bhopal, Bhopal, IND; 2 Anaesthesiology, All India Institute of Medical Sciences, Bhopal, Bhopal, IND; 3 Biochemistry, All India Institute of Medical Sciences, Bhopal, Bhopal, IND

**Keywords:** sofa score, disease activity, addisonian crisis, sepsis, rheumatological emergencies

## Abstract

Background: Autoimmune rheumatic diseases (ARD) present unique challenges in clinical practice. Many of them present in medical emergencies in an unstable state and need immediate evaluation for further plans of action. The clinical conundrum is to distinguish between sepsis, disease flare, or Addisonian crisis (AC) (secondary to steroid withdrawal). This may be further complicated by overlapping clinical features like shock/fever and the coexistence of a combination of the above pathophysiologic mechanisms (e.g. AC with sepsis or AC with disease flare). The known biomarkers may not perform optimally to distinguish them and additional supportive investigations like imaging, cultures, autoimmune serological markers, etc. are needed. Ultimately the boundaries between “the art of medicine” and “the science of medicine” may get blurred, as the established literature evidence falls short and the expert opinion is needed in a time-sensitive manner. In this pragmatic study, researchers have attempted to explore the presentation of rheumatologic emergencies on the above three differentials (sepsis, disease flare, and AC).

Materials and methods: In this hospital-based cross-sectional study, adult patients (age >18 years) with ARD who had unplanned hospital admission due to acute worsening were enrolled. This study was conducted over one year, after getting the Institutional Human Ethics Committee’s approval. All relevant hematological, immunological, and hormonal parameters (specifically morning cortisol) were collected and analyzed. The aim was to find the individual and combined prevalence of sepsis, disease flare, or AC in this study group.

Results: Forty-one patients were analyzed, with females in the majority (95%) and the dominant age group being 26-49 years (56.1%). A majority had a diagnosis of rheumatoid arthritis (RA) (56.1%) or systemic lupus erythematosus (SLE) (31.7%); the rest were other connective tissue diseases (12.2%). High-risk Quick Sequential Organ Failure Assessment score (qSOFA) score 2-3 was present in 29.3% while the rest had low-risk scores (qSOFA score 0-1). Thirty-two percent had severe disease activity, 46% had mild to moderate disease activity, and 22% of patients had no disease activity. While 78% of patients had low procalcitonin (PCT) values <0.5 microgm/L (low risk of sepsis), 15% had <20 microgm/L, and 7% percentage of patients had serum levels >20 microgm/L (high risk of sepsis). A total of 73.2% of patients had no evidence of infection while 26.8% had either microbiological/radiological evidence of infection. Only 7% of all patients had the presence of an AC. qSOFA scores didn’t statistically correlate with a diagnosis of infection or AC but positively correlated with PCT and C-reactive protein (CRP) values. Serum PCT didn’t correlate with the presence of infection with statistically significance (p-value 0.217).

Conclusion: Infections and sepsis are the most important considerations in the emergency presentations of ARDs. Disease flare and AC are also important differentials. Current inflammatory biomarkers like serum CRP and PCT may be less valuable for discriminating between infectious and non-infectious sepsis, especially in chronic inflammatory diseases like ARDs. qSOFA scores may have a prognostic role with less discriminant value. Management of ARD emergencies needs better biomarkers and more research is warranted.

## Introduction

Autoimmune rheumatic diseases (ARD) commonly present with medical emergencies. Medical emergencies may range from the flare of rheumatic complaints e.g. increased joint pain and stiffness to life-threatening conditions like septic shock/scleroderma renal crisis/catastrophic antiphospholipid antibody syndrome (APLA)/pulmonary hemorrhage, etc. In life/organ-threatening scenarios the most important differentials are an infection, acute disease flare, and Addisonian crisis (AC) [1,2]. It is extremely critical that an accurate diagnosis is made to do a timely intervention in such cases. However, it is not simple because quite often these co-exist and overlap. Other complications include drug toxicities or adverse reactions, anemia, or incidental events unrelated to ARDs [3,4].

Individuals with rheumatoid arthritis (RA) are recognized to be at increased susceptibility to infections [5]. The typical infections that are observed in individuals with RA are skin/soft tissue infections, septic arthritis, osteomyelitis, respiratory infection, intra-abdominal infections, urosepsis/pyelonephritis, severe gastrointestinal infections, etc. [6]. Modifications in the cellular immune system e.g. T cell capabilities, probably lead to higher infection risk. Many other risk factors for the occurrence of infection include additional comorbidities, leukopenia, age, chronic lung disorders, alcoholism, dementia/Alzheimer's condition, and diabetes mellitus. Therapy for RA - biologics, disease-modifying anti-rheumatic drugs (DMARDs), and corticosteroids, may also put individuals at increased risk for severe infection [7-11]. In contrast to the increased risk of infection associated with the use of corticosteroids and biologics, synthetic DMARDs don't seem to be connected with such a threat [12]. Two severity indices of RA like high erythrocyte sedimentation rate (ESR) and rheumatoid factor positivity tend to be predictive of the advancement of severe infection in RA patients. The risk of severe infection in patients with systemic lupus erythematosus (SLE) was also assessed and it was discovered that in SLE, 20-30% of deaths are induced by infection [13]. In a study, the risk of serious infections in SLE was roughly five times higher than in RA patients. In the subgroup of lupus nephritis (LN), this potential risk was nearly 12 times above that observed in RA patients [14]. Males and black populations with SLE have a heightened danger of severe infections. SLE's most typical severe infections are bacterial like pneumonia, urinary tract infections, opportunistic infections, sepsis, and skin infections [15]. If there is an underlying infection, clinicians must be vigilant to intervene early in sepsis with appropriate antibiotics and whenever the patient is on immunosuppressants, it’s an important decision to stop these immediately if possible and recommence after the infection is treated. However, it is easier to recommend than to practice clinically; as withdrawal of steroids, for example, may precipitate flare/AC and a judgment call is made to balance the risks.

The idea of disease activity is used for characterizing the disease severity and disease progression. Disease activity needs to be differentiated from illness severity, which happens to be a principle comprising wider facets of the disease progression and its various consequences. Symptoms of disease activity are reversible and stand as the primary goal of symptomatic treatment. Disease activity might be evaluated to characterize the present condition of the illness, in order to look at components of the patient's suffering; to attain a photograph of the fluctuating disease course; to keep track of the in-patient over time; to foresee additional outcomes and also to make choices about the therapy. Disease activity indices like Disease Activity Score 28 (DAS28), Simple Disease Activity Index (SDAI), and Clinical Disease Activity Index (CDAI), or maybe questionnaires, like Routine Assessment of Patient Index Data 3 (RAPID3), Patient Reported Outcomes - Clinical Arthritis Index (PRO-CLARA) and RA Impact of Disease (RAID), are utilized in RA [16]. Similarly, SLE Disease Activity Index (SLEDAI) is used in SLE [17]. These enable the doctor to know disease activity ranges as well as to monitor long-term response to therapy quickly and easily. These end results measures are able to assist in identifying the problems the patient is experiencing and will additionally help develop a far more extensive understanding of the individual’s improvement. An understanding of disease activity allows the doctor to determine whether to recommend alternative treatments or drugs. The modified DAS 28 score is most often employed to look at the disease activity in joints that are involved and is subsequently divided into lower, high, and moderate disease activity groups [18]. If there is an acute disease flare, then the clinician must decide to escalate immunosuppressants and steroids for a better outcome. Occasionally, disease flare in SLE may be difficult to distinguish from sepsis or AC.

RA is a chronic inflammatory autoimmune condition impacting the connective tissue structures surrounding the bones and adrenal function plays a crucial part. Adrenal stress hormones and their interactions with the body's immune system might affect the development and progression of symptoms of RA. Inflammatory pathogenesis, in turn, functions as a continual stress on the adrenals, and also could bring about adrenal fatigue. Additionally, it's tough to optimize endogenous adrenal function while administering powerful corticosteroids, and there is a lag period for adrenal functionality to completely rebound after an extended course of these drugs. It takes persistence and also the cautious assistance of an experienced doctor to effectively wean off corticosteroids and enhance adrenal function. Complete adrenal assistance could be an important help for individuals with RA [19]. Individuals taking long-term glucocorticoid therapy are in danger of acquiring AC during therapy [20]. The AC requires urgent diagnosis and intervention, and the treatment of patients who present in possible AC should not be delayed, to preventing mortality.

In this pragmatic and exploratory study, we aimed to estimate the prevalence of each complication (sepsis, AC, and disease flare) and potential overlaps. Adult Indian ARD patients presenting at a tertiary-level hospital in central India will be the study population. There is a lack of available literature focusing on the prevalence of the three mentioned presentations in the Indian context. Identifications of these complications by biomarkers and biochemical parameters may potentially simplify clinical management.

## Materials and methods

This observational study was performed at a tertiary-level hospital in central India. The study participants were enrolled after obtaining Institutional ethical clearance (AIIMS Bhopal Institutional Human Ethics Committee - IHEC - LOP/2019/MD0057 dated 27 April 2019) and written informed consent. Adult patients (age >18 years) with ARD who had an unplanned hospital admission due to acute worsening were enrolled over a period of one year. Planned admissions in rheumatologic care e.g. day-care admissions for biologics infusions, articular injections, etc. were not screened for inclusion in the study. Organ dysfunction was used as an indicator of acute worsening, and any of the patients satisfying one or more than one criterion were included in this study. Criteria were hypotension defined as systolic blood pressure <90 mm Hg or respiratory rate >22/minute or altered mental status defined by a Glasgow Coma Scale score <15 or new onset thrombocytopenia defined by a platelet count <1.5 lakhs/microL or new onset rise in creatinine >1.2 mg/dL or new onset rise in liver enzymes. Exclusions were ARD patients with intercurrent malignancy, not willing to give consent to or participate in the study and pregnancy. Available results of investigations performed as part of the standard of care of all participants, such as complete blood counts, ESR, renal function tests, liver function tests, arterial blood gas analysis, chest radiographs, blood culture/sensitivity, urine culture/sensitivity, viral serologies, morning serum cortisol, serum procalcitonin (PCT), C-reactive protein (CRP), urine routine and microscopy, urine albumin/creatinine ratio, and fasting blood sugar were collected. Specific investigations were collected e.g. computerized tomography scans/magnetic resonance imaging, sputum for acid-fast bacilli/cartridge-based nucleic acid amplification test (CBNAAT)/adenosine deaminase; echocardiography, anti-double-stranded DNA titers (anti-dsDNA)/creatinine kinase/lactate dehydrogenase/iron studies/bone marrow aspiration studies, etc. wherever available. Collected lab reports and data were entered in data extraction form. For the determination of acute systemic inflammatory response, Quick Sequential Organ Failure Assessment (qSOFA/SOFA) scores were used. Disease flare/uncontrolled disease activity was correlated with various laboratory parameters like C3/C4 complement levels (measured by nephelometry) and anti-dsDNA titers (measured by ELISA) (when applicable), and biochemical markers like CRP and ESR levels. Disease activity was measured using DAS 28 CRP values in RA and SLEDAI for SLE activity. For defining disease flare, in RA increase in DAS 28 score >1.2 points and in SLE >4 points on SLEDAI, from the last documented scores were taken as cutoffs. Microbiological confirmation of infections needed culture positivity or serological/antigen assays and radiological evidence with supportive investigations like sputum/urine examinations. Diagnoses of infectious causes of sepsis were correlated with various laboratory tests like PCT levels, and blood/urine cultures. Renal function tests, liver function tests, and arterial blood gas analysis was done to identify organ dysfunctions. Diagnosis of AC was done by morning serum cortisol concentration (less than 3 mg/dL (80 nmol/L)). Serum PCT was measured once within 24 hrs of hospitalization. It was quantitatively measured using fluorescent immunoassay.

The study variables were collected in a structured data collection form as either continuous or dichotomous variables. The data were entered in Microsoft Excel, and after data cleaning and analyzed using Statistical Product and Service Solutions (SPSS) (IBM SPSS Statistics for Windows, Version 16.0, Chicago) data analysis software. Analysis of the data was done on the basis of important statistical parameters like the mean, standard deviation, etc. The chi-square test was applied to determine the significance level of associations of AI, acute disease flare, and superimposed infection with the qualitative variables such as outcome, and various clinical parameters. We used a p<0.05 as a significance level for these comparisons.

## Results

Over one year period, a total of 78 patients with ARDs were admitted to medical wards. Sixty-three patients who satisfied the inclusion criteria during these periods were approached for inclusion, but 12 denied consent for participation. Of the remaining 51, another 10 were excluded because of insufficient information (unavailability of reports, mortality before blood investigations, etc). After these exclusions, data from 41 patients were analyzed.

Demographically, in this study majority were in the age group 26-49 years (56.1%) and were females (95.1%). A majority had a diagnosis of RA (56.1%) or SLE (31.7%); the rest were other connective tissue diseases (primary Sjogren, mixed connective tissue disease, etc). A majority (63%) of patients had a duration of hospital stay of more than five days and in-hospital mortality was about 22%.

The qSOFA score is a validated predictor of mortality in patients with suspected sepsis [21]. A total of 46.3% of all patients had a qSOFA score value of zero, 24.4% of patients had q SOFA 1, 24.4% of patients had a qSOFA score of 2, and 4.9% had a qSOFA score of 3. About one-third of patients had high disease activity while about one-fifth had no disease activity. Serum CRP, an acute phase reactant, was normal (<5 mg/dL) in about 46% and a minority (17%) had high values >50mg/dL. Positive serum CRP (>5 mg/dL) was significantly associated with the presence of disease activity (p-value<0.0001). Serum PCT levels were low (<0.5 microgm/L) in 78% of patients (low risk of sepsis); 15% had < 20 microgm/L, and 7% of patients had serum levels > 20 microgm/L (high risk of sepsis). Serum PCT values didn’t correlate with the presence of infection (p-value 0.217) (Table 1).

**Table 1:** Correlation of PCT with evidence of infection PCT: procalcitonin

			Evidence of infection		Total
			Present	Absent	
Serum PCT	Low (<0.5 microgm/L)	Count	4	5	9
		% within Serum PCT	44.4%	55.6%	100.0%
	High (>0.5 microgm/L)	Count	7	25	32
		% within Serum PCT	21.9%	78.1%	100.0%
Total		Count	11	30	41
		% within Serum PCT	26.8%	73.2%	100.0%

Only about 7% of patients had evidence of AC based on low early morning serum cortisol levels (<3 mg/dL (80 nmol/L)). An adrenocorticotropic hormone (ACTH) stimulation test was not performed. In terms of the association of qSOFA scores with complications in ARD, no significant statistical association was found with a diagnosis of infection or AC but the score was significantly associated with serum PCT (p-value 0.02) and CRP values (p-value <0.0001) (Table 2).

**Table 2:** Correlation of qSOFA scores with serum PCT values PCT: procalcitonin; qSOFA: quick Sequential Organ Failure Assessment score

			Serum PCT		Total
			Low (<0.5 microgm/L)	High (>0.5 microgm/L)	
qSOFA	Zero	Count	0	19	19
		% within qSOFA	0.0%	100.0%	100.0%
	More than or equal to one	Count	9	13	22
		% within qSOFA	40.9%	59.1%	100.0%
Total		Count	9	32	41
		% within qSOFA	22.0%	78.0%	100.0%

Among nine patients with death as an outcome, qSOFA was elevated in all the patients; AC was present in 11%; evidence of infection was present in 44%; disease flare and elevated CRP was found in all the patients (Table 3).

**Table 3:** Characteristics in patients with mortality qSOFA: quick Sequential Organ Failure Assessment score; CRP: C-reactive protein; PCT: procalcitonin

	Present	Absent
qSOFA score (>1)	9	0
Addisonian crisis	1	8
Evidence of infection	4	5
Disease activity (moderate or high)	9	0
Serum CRP (>5mg/dL)	9	0
Serum PCT (<0.5microgm/L)	6	3
Duration of hospital stay (>5 days)	8	1

## Discussion

ARDs presenting in emergencies have unique challenges; they have an underlying predisposition for infections, increased further by immunosuppressive treatment. Additionally, they may have increased disease activity and AC. With no gold standard biomarkers or biochemical markers distinguishing coexisting disease activity from sepsis, its management may be tricky.

RA patients most frequently present in emergencies, probably reflecting their high prevalence in population and chronicity. A study conducted in a rheumatology clinic in Turkey among 2700 ARD patients during a period of four years between 2014 and 2018 found that patients who visited the emergency department were generally middle-aged females (mean age 44 years). Additionally, among the patients who had visited the emergency department, the majority had RA, which is the most common inflammatory rheumatic disease worldwide [22]. In our study, 95% of patients were females and around 56% of patients were in the age group 26-49 years.

Sepsis is one of the most common emergency presentations in ARDs. Forty deaths were reported out of an Indian cohort of 329 SLE patients [23]; 16 of which were due to renal failure; eight were due to infection; eight were due to acute uncontrolled SLE; and eight were due to neurological involvement. The risk of serious infections was higher in RA compared with those patients with non-inflammatory rheumatic musculoskeletal diseases [24]. In our study, 26.8% of patients had either microbiological or radiological evidence of infection.

AC may present as a clinical mimic for sepsis and disease flare. In a critical care unit study, secondary adrenal insufficiency was seen in 31% of patients [25]. In our study, 7% of all patients and 11% of patients with mortality had AC. In this study, around 36% of patients with elevated qSOFA scores had the presence of infection; though the relationship between qSOFA values and evidence of infection was not statistically significant. More than 80% of patients with high CRP had elevated qSOFA values. Similarly, it was shown that there is a positive significant relationship between qSOFA values at admission and serum PCT (p-value 0.02). The relationship between qSOFA values at admission and outcome at the end of hospital stay in patients was also studied and it was concluded that patients with q SOFA values <1 at admission are more likely to have a better outcome. Serum PCT is widely debated as a biomarker for the early identification of bacterial infections. It is evolving as a promising marker for the early diagnosis of sepsis in critical patients [26], it is also useful for clinicians when a decision of de-escalation or discontinuation of antibiotics is being made [27]. It thus reduces unwanted antibiotic exposure and may prevent resistance [28]. In light of this, we tried to study the relationship between serum PCT and evidence of infection, though no statistically significant relationship was observed. Serum PCT may be less useful as a biomarker of bacterial infections, in chronic inflammatory disorders.

The limitation of this study is the small sample size, which may not be adequate to extrapolate our results to the general population. The majority of our study population were RA and SLE patients, with other autoimmune diseases being less represented. ARDs are itself a highly heterogenous group and many undiagnosed ARDs presenting in emergencies might have been excluded. Hence our findings cannot be extrapolated to all rheumatological conditions. This study is a pragmatic analysis of ARD emergencies.

## Conclusions

Infections and sepsis are the most important considerations in the emergency presentations of ARDs. Disease flare and AC are also important differentials. Current inflammatory biomarkers like serum CRP and serum PCT may be less valuable for discriminating between infectious and non-infectious sepsis, especially in chronic inflammatory diseases like ARDs. qSOFA scores may have a prognostic role with less discriminant value. Management of ARD emergencies needs better biomarkers and better methodologies to characterize the pathological state.
